# Modules, networks and systems medicine for understanding disease and aiding diagnosis

**DOI:** 10.1186/s13073-014-0082-6

**Published:** 2014-10-17

**Authors:** Mika Gustafsson, Colm E Nestor, Huan Zhang, Albert-László Barabási, Sergio Baranzini, Sören Brunak, Kian Fan Chung, Howard J Federoff, Anne-Claude Gavin, Richard R Meehan, Paola Picotti, Miguel Àngel Pujana, Nikolaus Rajewsky, Kenneth GC Smith, Peter J Sterk, Pablo Villoslada, Mikael Benson

**Affiliations:** Centre for Individualized Medicine, Department of Pediatrics, Faculty of Medicine, 58185 Linköping, Sweden; Department of Physics, Biology and Computer Science, Center for Complex Network Research, Northeastern University, Boston, MA 02115 USA; Department of Neurology, University of California, San Francisco, CA 94143 USA; Center for Biological Sequence Analysis, Department of Systems Biology, Technical University of Denmark, DK-2800 Lyngby, Denmark; Novo Nordisk Foundation Center for Protein Research, Faculty of Health Sciences, University of Copenhagen, DK-2200 Copenhagen, Denmark; Airways Disease Section, National Heart and Lung Institute, Imperial College London, London, SW3 6LY UK; Department of Neurology and Neuroscience, Georgetown University Medical Center, Washington, DC 20057 USA; European Molecular Biology Laboratory, 69117 Heidelberg, Germany; MRC Human Genetics Unit, MRC IGMM, University of Edinburgh, Edinburgh, EH4 2XU UK; Institute of Biochemistry, University of Zürich, 8093 Zürich, Switzerland; Catalan Institute of Oncology, Bellvitge Biomedical Research Institute (IDIBELL), Barcelona, 08908 Spain; Systems Biology of Gene Regulatory Elements, Max-Delbrück-Center for Molecular Medicine, Robert-Rössle-Strasse 10, 13125 Berlin, Germany; Cambridge Institute for Medical Research, University of Cambridge, Cambridge Biomedical Campus, Cambridge, CB2 0XY UK; Department of Medicine, University of Cambridge School of Clinical Medicine, Addenbrooke’s Hospital, Cambridge, CB2 0QQ UK; Department of Respiratory Medicine, Academic Medical Centre, University of Amsterdam, 1100 DE Amsterdam, The Netherlands; Center of Neuroimmunology and Department of Neurology, Institut d’investigacions Biomèdiques August Pi i Sunyer (IDIBAPS), Hospital Clinic of Barcelona, 08028 Barcelona, Spain

## Abstract

Many common diseases, such as asthma, diabetes or obesity, involve altered interactions between thousands of genes. High-throughput techniques (omics) allow identification of such genes and their products, but functional understanding is a formidable challenge. Network-based analyses of omics data have identified modules of disease-associated genes that have been used to obtain both a systems level and a molecular understanding of disease mechanisms. For example, in allergy a module was used to find a novel candidate gene that was validated by functional and clinical studies. Such analyses play important roles in systems medicine. This is an emerging discipline that aims to gain a translational understanding of the complex mechanisms underlying common diseases. In this review, we will explain and provide examples of how network-based analyses of omics data, in combination with functional and clinical studies, are aiding our understanding of disease, as well as helping to prioritize diagnostic markers or therapeutic candidate genes. Such analyses involve significant problems and limitations, which will be discussed. We also highlight the steps needed for clinical implementation.

## The complexity of common disease

Despite impressive advances during the past century, modern health care is faced with enormous challenges. One problem is that currently available drugs show highly variable clinical efficacy, which results not only in suffering, but also contributes to increasing costs. The annual cost of ineffective drugs in the US alone is estimated at US$350 billion [1]. Variable efficacy also adds to the huge costs associated with drug discovery, development and clinical trials (on average US$1 billion per drug), which further impacts the financing of health care. These problems reflect the complexity of common diseases, which can involve altered interactions between thousands of genes. Because of the large number of genes and their interconnection, it is very difficult to gain functional understanding of disease mechanisms by detailed studies of individual genes.

**Figure 1:**
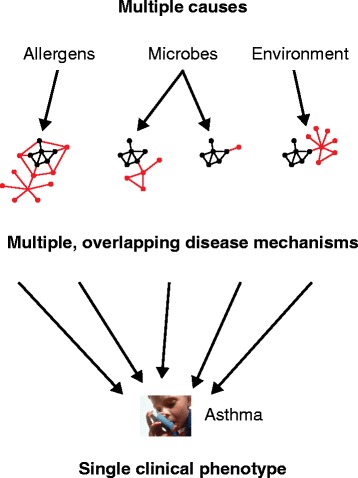
**A single disease phenotype can be caused by multiple mechanisms.** As an example, asthma can be triggered by allergens, microbes and other environmental factors, each of which may activate different disease mechanisms, which are depicted as shared (black) and specific (red) networks.

This problem of complexity is compounded by disease heterogeneity: patients with similar clinical manifestations may have different underlying disease mechanisms. Asthma is an example of such a disease; it can be caused by infection, allergens or other environmental factors, which give rise to different inflammatory responses (Figure 1). Variations in response may underlie the observation that between 10 and 20% of patients do not respond to one of the most common asthma drugs, corticosteroids [2]. This variation, however, can potentially be exploited to find novel drugs for nonresponders in asthma, allergy and other diseases, as well as to identify patients that require such drugs [3].

Despite the success of single diagnostic markers, there is a pressing need for multiple markers. Single markers are already being used in the clinic to predict disease or personalize treatment and examples include BRCA genotyping in breast cancer, CCR5 mutation status in HIV infection and newborn screening for metabolic defects [4]. Recently, optimization of the anticoagulant therapy warfarin based on genotyping of two genes was described [5]. However, the diagnostic accuracy of individual or pairs of biomarkers is likely to be limited as only a fraction of disease-associated genes is predicted to have a large effect on any specific disease; most disease-associated genes have small effects [6]. Yet, the combined effect of these small-effect genes may be large. Thus, the accuracy of a biomarker based on a large-effect gene may vary depending not only on variations in that gene, but also on variations in the many genes with small effects.

Systems medicine is an emerging discipline that aims to address the problem that a disease is rarely caused by malfunction of one individual gene product, but instead depends on multiple gene products that interact in a complex network [7]. Here, we explain how and why systems medicine, and specifically network approaches, can be used to assist clinical decision making and to identify underlying disease mechanisms. We focus on the use of disease modules to uncover pathogenic mechanisms and describe how these can be extended into multilayer networks. We finish by discussing the current problems and limitations of network and systems approaches and suggest possible solutions. We also highlight the necessary steps for clinical implementation. We focus on systems medicine as a network-based approach to analysis of high-throughput and routine clinical data to predict disease mechanisms to diagnoses and treatments.

## Systems and network medicine to support clinical decision-making

Similar to many evolving medical disciplines, there is no generally accepted definition of systems medicine, although different proposals are available [8,9]. Some view it as an interdisciplinary approach that integrates research data and clinical practice and others view it as fusion of systems biology and bioinformatics with a focus on disease and the clinic. Recent articles have described systems medicine as a high-precision, mathematical model of variables from different genomic layers that relate to clinical outcomes such as treatment response [10,11]. Rather than trying to distinguish between systems medicine and other disciplines, our review is based on the premise that systems medicine is a natural extension of, or is complementary to, current models for clinical decision-making.

In general, clinical decisions are based on a diagnostic model consisting of multilayered pattern recognition of multiple data inputs linked to scientific reasoning about causality. This diagnostic model can be exemplified by pneumonia. On a phenotypic level, pneumonia is often characterized by fever and symptoms or signs of changes in the respiratory tract. This layer of information can be linked to data (such as radiographic imaging, laboratory tests for inflammatory signs of infection and microbial tests) that suggest the cause of the disease. The physician may need to take into account other layers, including socioeconomic and environmental factors. For example, if the patient is homeless and a smoker, this is likely to affect diagnosis, treatment and the innate immune response of the patient to the infection. Thus, in the case of pneumonia, accurate diagnostic decisions can be made by pattern recognition and reasoning.

However, for many diseases, diagnosis is more difficult. The external causes, disease mechanisms or the involvement of cells, tissues or organs may be highly complex or only partially known. In such cases, the physician would be helped by a formal diagnostic model that gave decisional support by presenting the variables so that contributory disease mechanisms can be elucidated and diagnostic predictions computed. One approach is to use a template in which omics clinical variables are organized into a network to understand disease mechanisms and make diagnostic predictions. Such a template would naturally build on the current diagnostic model of pattern recognition. Using this diagnostic model would allow different clinical variables, such as symptoms and laboratory variables, to be described in different network layers. In this way, multilayer network models can be constructed that include all known relevant variables, ranging from genetic variants to environmental factors.

In summary, the potential advantage of a multilayer network model is that it provides a framework in which to organize and analyze all relevant disease data simultaneously, thereby informing and improving the decisional pathway of medical professionals and patients [12]. Before we look at how networks and modules can be used to uncover disease mechanisms, we first provide an overview of networks in biology.

## A brief introduction to networks

Networks provide graphical representations of complex systems. In the context of cellular networks, molecules such as genes and proteins are represented as nodes, and the interactions among them as links. In a landmark article in 1999, it was shown that networks in technological, social and biological systems have common designs that are governed by simple and quantifiable organizing principles [13]. Key findings were that a fraction of the nodes serve as hubs with multiple links, whereas the vast majority of nodes have few links. The hubs often have large individual effects, in contrast to the nodes with few links. The hubs contribute to the small world property of networks: all nodes in a network are generally connected by a limited number of links. Another important characteristic is that functionally related nodes tend to be highly interconnected and co-localize in networks, thereby forming modules [7,14] (Table 1).

**Table 1:** **Glossary of terms**

**Term**	**Description**
Network	A graphical representation of a complex system. For example, in a protein network, proteins are nodes, and interacting proteins are linked by edges
Disease module	When mapped onto the protein-protein interaction network, disease-associated genes tend to co-localize and form networks of functionally related genes. These networks are referred to as disease modules
Multilayer disease	A module whose nodes and edges are located across different layers of disease-relevant information. Such layers could include transcription factor networks, genetic variants and even environmental factors

In the context of disease, disease-associated genes identified by omics studies can be computationally mapped on to models of the human protein-protein interaction (PPI) network. In other words, each disease-associated gene is mapped on to its matching protein product. The resulting maps have characteristics that are similar to those found in other types of networks. One of the most important characteristics is that functionally related genes tend to co-localize and form disease modules.

## Disease modules for understanding pathogenic mechanisms

**Figure 2:**
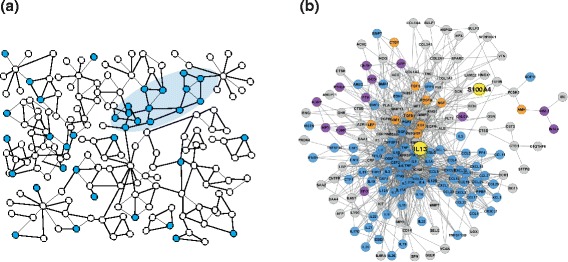
**A disease module. (a)** Conceptual model of how disease-associated genes (blue nodes), identified by high-throughput analysis, tend to co-localize in the human protein-protein interaction network (white nodes), forming a module (blue oval). The genes in the module are assumed to be more important for the disease than extramodular genes. **(b)** An actual disease module from allergic patients, showing extracellular proteins that were putatively co-regulated with IL13. Blue nodes are associated with cytokine activity, purple nodes are associated with hormone activity, and orange nodes are associated with growth factor activity according to Gene Ontology Molecular Function. The diagram in (b) is reproduced, with permission, from Bruhn *et al. Science Translational Medicine* 2014 [33].

Disease modules can help to organize and prioritize disease-associated genes identified by high-throughput analyses (Figure 2), as well as to provide an overview of disease mechanisms by performing pathway analyses. Disease modules can also help to identify novel disease genes, biomarkers or therapeutic targets. Remarkably, one landmark study for systems medicine was initiated by researchers without a clinical background, who had studied network design principles in model organisms like yeast cells or worms [15]. In 2007, Pujana *et al*. [16] described a module relevant to breast cancer, and identified a novel candidate gene, *HMMR*, that was validated by functional and genetic studies. Several module-based studies have been performed in other diseases, including cancer [17-20], neurological [21-23], cardiovascular [24], and inflammatory diseases [25-27]. One of the studies showed how protein interaction modules could be used to predict outcome in breast cancer [20]. In a study of autoimmune diseases, mRNA modules were used to predict disease progression based on functional studies of underlying mechanisms [28]. In 2014, a module-based approach for drug discovery was described in rheumatoid arthritis based on a meta-analysis of genome-wide association studies (GWASs) of 100,000 subjects [29].

Analysis of disease modules exploits the general principles of networks, such as alteration of hub genes being likely to have large effects, while alterations in the many genes with few links will likely correspond to small-effect genes. Thus, specific therapeutic targeting of a hub gene is more likely to be effective than targeting a gene with few interactions. Indeed, genes targeted by drugs have more interactions than other genes [30], which increases the risk that a drug targeting a specific disease gene may have an off-target effect [31]. An important observation is that nodes that are highly interconnected in a network are likely to be functionally related. Thus, novel candidate genes can be found among the interactors of known disease genes [32].

**Figure 3:**
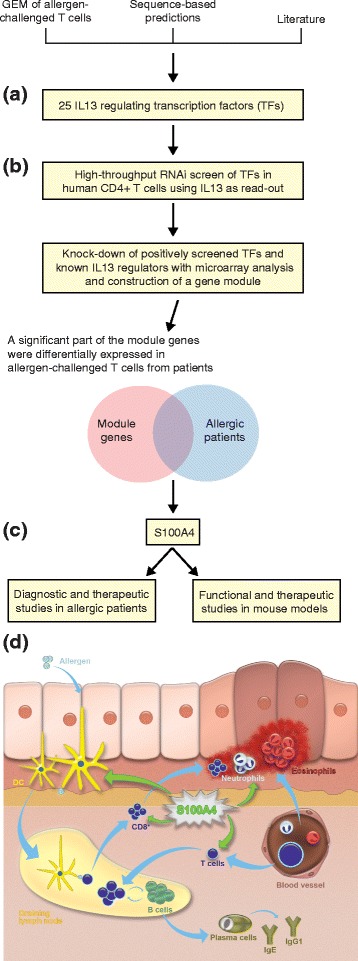
**A module-based approach to identify disease-relevant diagnostic and therapeutic candidate genes in allergy. (a)** Twenty-five putative *IL13*-regulating transcription factors (TFs) were identified by combining data from mRNA microarrays, sequence-based predictions and the literature. **(b)** *IL13*-regulating TFs were validated by siRNA-mediated knockdown of the 25 TFs in human total CD4^+^ T cells polarized toward T_H_2 using *IL13* as a read-out. The target genes of the TFs were identified by combined siRNA knockdown of the positively screened TFs/known *IL13*-regulating TFs from literature and microarray analyses. This resulted in a module of genes that was co-regulated with *IL13* in T_H_2-polarized cells and significantly overlapped with differentially expressed genes from allergen-challenged T cells from allergic patients. For further validation experiments, the study focused on module genes that encoded secreted proteins and had not been previously associated with allergy. **(c)** Functional, diagnostic and therapeutic studies involving one of the module genes, *S100A4*, were performed in patients with seasonal allergic rhinitis, allergic dermatitis and a mouse model of allergy. **(d)** Model of S100A4-induced disease mechanisms. Allergic inflammation requires the sensitization of the immune system by allergens, resulting in the production of antigen-specific T cells. The interaction of dendritic cells (DC) in the draining lymph node with T cells is a critical step that is dependent on S100A4. B-cell maturation as a result of T cell-B cell crosstalk (for example, the release of T_H_2 cytokines by T cells) leads to the production of IgE and IgG1 by plasma cells. Cytokines and chemokines released by T cells stimulate the migration of circulating granulocytes (for example, neutrophils and eosinophils) to the inflammatory site (skin). Differentiation of naïve T cells into CD8^+^ cytotoxic T cells will exacerbate the skin damage. Blue arrows indicate the flow of the allergic responses. Green arrows indicate the promotion of these processes by S100A4. GEM, gene expression microarray.

One recent example of a successful module-based approach was based on the assumption that the genes in a module would be co-regulated by the same set of transcription factors (TFs) that regulate a known disease gene, *IL13* [33] (Figure 3). Twenty-five putative *IL13*-regulating TFs were knocked down using short interfering RNA (siRNA), of which seven were found to affect *IL13*. The knockdowns were repeated for these TFs, followed by mRNA microarrays to detect their downstream targets. This led to the identification of a module of highly interconnected genes. That module contained several genes of known relevance to allergy, such as *IFNG*, *IL12*, *IL4*, *IL5*, *IL13* and their receptors. It also contained novel candidate genes, including *S100A4*, which was validated as a diagnostic and therapeutic candidate by a combination of functional, mouse and clinical studies. A mouse knock-out model showed that *S100A4* had extensive phenotypic, cellular and humoral effects on allergic inflammation. The therapeutic potential was demonstrated by treatment with a specific antibody, both in the mouse model and in cells from allergic patients.

## Multilayer disease modules

The success of single module approaches in identifying candidate genes prompted researchers to extend it to multiple modules to link genomic, phenotypic and environmental variables together. Rapid development of high-throughput techniques has enabled global analyses of different network layers ranging from DNA to proteins, as well as metabolites and lipids [34,35]. Similar to genes, the variables in each layer can be linked to each other. Consider, for example, one disease module formed by mRNAs and another from single nucleotide polymorphisms (SNPs). If an mRNA and a SNP in each module map to the same protein, they can be linked. This principle can be expanded to all proteins in the module and the overlap tested statistically. Another example is modules formed by genes and their regulators, such as TFs or microRNAs. Genes can be linked if they are regulated by the same microRNAs, and a double-layer module can then be formed by linking microRNAs that regulate the same gene. By combining different high-throughput analyses it is therefore possible to form multilayer disease modules (MLDMs).

Multidimensional models can be used to form rejectable hypotheses of how genes, gene products and regulators interact with each other. For example, does a disease-associated SNP in a promoter region of a module gene change the expression of that gene? Does a microRNA regulate its predicted target genes in a module? The clinical relevance of MLDMs lies in that they can provide a framework to identify optimal combinations of diagnostic markers from different layers, based on functional understanding of the pathogenic roles of those markers. For example, microRNAs and genetic variants have been used to examine disease-associated variations in mRNA expression in gliomas, and to predict disease outcome [36,37]. In allergy, functional studies showed that mRNA modules were co-regulated by microRNAs, some of which had hub-like functions and potential diagnostic relevance [38].

**Figure 4:**
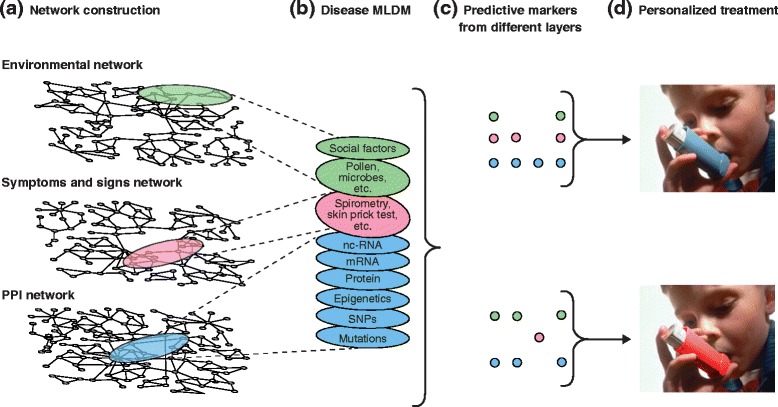
**An idealized systems medical approach to personalized treatment. (a)** All factors that influence a disease can potentially be described by networks. For example, symptoms and signs that tend to co-occur can be linked and form a module that corresponds to a disease (pink oval). That module may be linked to underlying modular protein changes (blue oval). Similarly, the disease module may be linked to co-occurring environmental factors (green oval). **(b)** Each of the modules in (a) can be further divided to represent different sublayers, from which **(c)** predictive markers from the different sublayers can be identified, and used for **(d)** personalized treatment. MLDM, multilayer disease module; nc-RNA, noncoding RNA; PPI, protein-protein interaction; SNPs, single-nucleotide polymorphisms.

An important aspect of MLDMs is that they can be linked to modules formed by other clinical data. For example, a link can be placed between a disease and a gene associated with that disease [39]. Next, diseases that are associated with the same gene can be linked and form a human disease network. The same principle can be applied to the disease genes forming a disease gene network. Such networks are modular and can be linked, so that diseases can be associated with the underlying disease mechanisms. It is also possible to construct and link modules containing other relevant data, such as social and environmental factors (Figure 4). It is of note that the construction of MLDMs is complicated by several technological limitations, which are discussed later in this review.

MLDMs might also be useful for tracking disease over multiple time points. Diseases are dynamic processes rather than static entities, and the underlying processes and time frames may range from hours in rapidly evolving cases, such as meningitis, to decades in cancer. Disease progression is perhaps best understood in cancer. For example, at a molecular level, a study of chronic lymphocytic leukemia revealed the development of substantial genetic heterogeneity of tumor cells from the same patients over time [40]. Such developments were linked to disease deterioration and variable treatment response. In breast cancer, module kinetics has been directly linked to treatment response; in a subset of patients, treatment with one drug rewired the disease module so that it became sensitive to another drug [41]. Thus, understanding of module kinetics can be exploited for sequential treatment with different drugs. Ideally, this principle should be expanded so that all diseases are staged using MLDMs with omics and routine clinical data integrated. In the future, it may be possible to infer early MLDMs, before patients become symptomatic, allowing preventative medicine.

It is possible that personal MLDMs could become a cornerstone for health care, and could be used for the early diagnosis of changes in module function, based on functional understanding of why disease-causing nodes in the MLDMs change (such as due to a genetic variant). As the bioinformatics principles for analyzing different forms of variables are largely the same, MLDMs could also include other forms of clinical information, such as routine laboratory tests and medical imaging. The versatility and resolution of medical imaging is steadily increasing and is aiming to provide functional understanding of observed structural changes in the human body. This would allow, for example, specific traits imaged in liver cancer to be linked to prognostic gene expression changes [42]. Similarly, obesity traits could be linked to molecular changes [43].

In summary, MLDMs can potentially be used as templates to integrate and analyze multiple layers of disease-relevant information. Similar to the current diagnostic model discussed above, analyses can be based on functional understanding, but with higher resolution and the option for computational predictions. When the underlying mechanisms are revealed, our view of various common diseases might alter, prompting reclassification of multiple diseases.

## Networks to reclassify diseases based on pathogenic mechanisms

The current diagnostic classification is based on observations of symptoms and signs, associations with external factors (for example, pollen and allergy), and use of diagnostic aids like radiology, and variable molecular knowledge of disease mechanisms. A fundamental problem with this classification system is that the same phenotype may result from multiple disease mechanisms. Thus, if a drug is only effective against one of those mechanisms, its use in patients with different underlying mechanisms will not be therapeutically successful.

**Figure 5:**
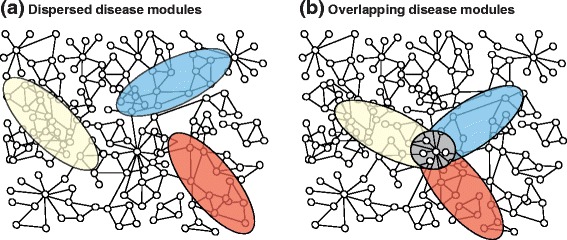
**Relationship between different disease modules on the protein-protein interaction network. (a)** A hypothetical model of three different diseases mapped on the human protein-protein interaction network. The modules are dispersed in the network. **(b)** Instead, meta-analysis of mRNA microarray and genome-wide association study data show that disease modules partially overlap and form a shared module (grey) [39]. The shared module has important pathogenic, diagnostic and therapeutic implications.

Ideally, diagnoses should be based on accurately linking phenotypes with all possible underlying mechanisms. Taking this idea to its extreme would require simultaneously analyzing all possible external causes and mechanisms. Since there is considerable comorbidity, all diseases should also be simultaneously analyzed. Actually, the first steps in this direction have been already taken, using network-based analyses of public databases and high-throughput data. In a landmark study, Goh *et al*. [44] mapped human disease genes onto the interactome, and found that genes associated with phenotypically similar diseases tended to co-localize. Similar observations were made for networks derived from expression profiling [45]. This led Barrenas *et al*. [39] to construct a module-based map of human diseases. Similar to a geographical map, different disease categories should co-localize in different parts of the interactome (Figure 5a). Ideally, such a map could be used as a reference to improve diagnostic accuracy and classification, and better identify diagnostic and therapeutic candidates. However, despite the diseases being very diverse (including metabolic, inflammatory and oncological diseases), they partially overlapped. Thus, instead of being dispersed in the interactome, the disease modules formed a flower-like structure (Figure 5b). The overlapping disease modules formed a new, shared module with remarkable characteristics. It was enriched for inflammatory, metabolic and proliferative pathways. Since these pathways have key roles in survival, this led to the hypothesis that altered function in one of the pathways may spill over to the others and cause one or more diseases. Indeed, meta-analysis of GWASs representing more than 100 diseases and hundreds of thousands of patients showed that the shared module was highly enriched for SNPs from these diseases [39]. These findings contrast with the dogma that diseases are mainly caused by disease-specific genes, and that nonspecific genes are secondary or irrelevant. Further studies showed that the shared module was more enriched for GWAS genes than disease-specific genes. Moreover, it was highly enriched for known biomarkers and therapeutic targets. Clinical studies showed that the expression profile of the shared module had the potential to stratify allergic patients for treatment with corticosteroids. Because the shared module was highly enriched for GWAS genes it is likely that it has an important causal role, which has diagnostic implications for predictive and preventative medicine [3,39].

Other approaches to disease reclassification have involved mining of electronic health records to search for comorbidity patterns and underlying genetic variants [46-51]. For example, by combining electronic health records and GWASs, Denny *et al*. [46] showed novel associations. For example, the presence of polymorphisms in *IRF4* was linked to skin cancer and actinic keratosis [46]. There are also enormous resources of biomedical relevance available in the public domain that can be analyzed with network-based principles. For example, Medline contains some 20 million abstracts, the Gene Expression Omnibus one million expression experiments, and the Encyclopedia of DNA Elements (ENCODE) more than 2,500 high-throughput experiments. In one study, new indications for known drugs were predicted based on integration of public expression data of more than 100 diseases and expression data from the drugs. For example, an antiulcer drug, cimetidine, was shown to be a therapeutic candidate in lung cancer [52]. In another study, a hypothesis about T-cell differentiation was tested completely *in silico*, by mining and modeling data in the public domain. All abstracts in MedLine were mined to construct a module relevant for T-cell differentiation. This module was tested by simulated activation and knockdown of individual module genes. The simulation yielded unexpected results, which were validated by analyses of correlation patterns in public mRNA microarray data from different T-cell-associated diseases [53]. It is likely that network-based analysis of highly diverse data sets with increasingly powerful computational tools will contribute to a new disease taxonomy. Already, there are examples of this, such as in severe asthma [54].

## Problems, limitations and opportunities

Every step of a systems medicine study, including the use of network and module approaches, involves problems and limitations. One problem is that high-throughput analyses often require large sample sizes to obtain statistically significant results, and sufficient samples may be difficult to obtain. In some diseases, it is difficult or impossible to obtain relevant clinical samples, such as neurodegenerative diseases. One solution to this problem, at this stage, may be to focus on particularly tractable diseases. As an example, in seasonal allergic rhinitis, the key external trigger (pollen) and the key cell type (lymphocytes) are both known and readily accessible. The disease occurs at a known time point each year. Thus, it is possible to mimic the disease process by *in vitro* challenge of T cells from patients outside of the pollen season. It is also possible to perform functional studies of candidate genes in activated T cells, or in a well-defined mouse model of allergy. The disease process and diagnostic markers can be analyzed locally in the affected organ [33].

Another issue is that many different cell types are often involved in one disease, and more than one may be important. The involvement of multiple cell types in the development of a disease introduces an additional challenge to the generation of meaningful MLDMs from omics data relying on cell mixtures. This may be addressed in the near future by the application of single-cell analysis technologies. Recent developments in sequencing allow determination of single-cell genomes and transcriptomes [55,56], while mass cytometry enables the targeted quantification of proteins and their modifications in different cells from a heterogeneous population [57].

Other challenges arise from technical problems, which include variation in the accuracy and sensitivity of high-throughput techniques. This is particularly so for global protein profiling, which is complex and difficult to perform in a clinical setting. The occurrence in a proteome of various post-translational modifications, SNPs and alternative splicing of proteins further complicates such analyses. However, recent technological advances indicate that targeted proteomics may partly address these limitations and render the analysis of predetermined sets of proteins over large numbers of samples [58,59]. Targeted protein assays may also enable the quantification of highly homologous protein sequences, such as splice variants, protein isoforms and mutated versions of a protein [60], in a clinical laboratory setting. Another emerging targeted proteomic application is the generation of perpetually reusable digitalized maps of the proteomic signals of a sample [61]. The thus generated maps can then be mined using targeted data extraction strategies to quantify disease-related proteins of interest over large cohorts of patient samples. Literature knowledge and MLDM layers that are more easily measured than proteins, such as mRNA or genomic information, could help to identify proteins for such targeted analyses [62]. Similarly, recent technical advances may help to include targeted metabolites and lipids in the MLDMs [63,64].

The bioinformatics analyses involve several problems of their own. For example, important limitations of PPI networks are that they are generally not cell specific, and are constructed based on heterogeneous sources such as literature and databases, experimental data, inferences from high-throughput studies, or computational predictions [65].

A key remaining problem is how to validate results from analyses involving thousands of genes or gene products. Systems medicine is based on combining genome-scale validation strategies with detailed studies of individual factors. Therefore, it is mandatory to follow recommendations for multiscale analysis [66], thereby strictly limiting false discovery [67]. Recently, these analyses have been anchored to MLDMs, by providing stepwise criteria for the use of omics-based predictors in clinical trials [68].

On a genomic scale, an important validation principle is to test for genomic concordance. In other words, to test if there is concordance between different layers in an MLDM. For example, it is possible to validate by examining if disease modules that are derived from mRNA microarray analyses are enriched for SNPs identified by independent GWASs of the same diseases. Another form of genome-scale validation is to examine if siRNA-mediated knockdowns of predicted upstream genes in a module result in altered expression of downstream module genes. If these two genome-scale analyses support the findings, then detailed functional and clinical studies can be performed, including mouse disease models [33].

## Clinical implementation of systems and network medicine

There are already examples of gene testing being used in the clinic. Diagnostic products to stratify breast cancer based on gene expression profiling are commercially available, such as the MammaPrint [69]. MLDMs could also be used to stratify patients for individualized medicine based on functional understanding of why patients do or do not respond to a particular drug. This could, in turn, lead to development of novel drugs for nonresponders, directed against mechanisms not targeted by existing drugs. MLDMs could also be used for repositioning of drugs that have not reached the market because of low efficacy or side effects.

The clinical implementation of systems medicine would require extensive clinical, administrative and educational adaptations. One current problem is that very few clinicians are involved in systems medical research, education or implementation. Yet, systems medicine is beginning to become a part of the curricula of many medical schools (for example, http://gumc.georgetown.edu/spi/systemsmedicine).

The European Commission has launched a project aiming to draw up a road map for the clinical implementation of systems medicine (https://www.casym.eu). This road map is based on integrating the views from different relevant stakeholders, including clinicians, basic researchers, representatives of the pharmaceutical industry, funding bodies and government health agencies. Educational programs for the training of health professionals at different stages of their careers, starting from medical school, have already started in the USA and some European countries.

It is important to recognize that systems medical principles are in line with clinical reasoning, and perhaps can be seen as a natural extension that permits formalized reasoning about pathogenic mechanisms, as well as diagnostic predictions.

## Conclusions and future directions

Many of the main challenges facing modern health care arise from the complex and heterogeneous characteristics of common diseases. The same phenotype may result from different mechanisms, and each mechanism will require a different treatment. Ideally as many phenotypes, genes and other disease-associated variables as possible should be studied together in order to reclassify diseases based on functional understanding of underlying mechanisms. However, this involves multiple, large-scale methodological, economical and ethical challenges, which are only partially resolved.

The rapid development of systems medicine is illustrated by a prospective study of 100 healthy subjects, known as the Hundred Person Wellness Project, which was started in March 2014. Blood, urine and stool samples will be regularly analyzed for multiple biomarkers or microbes, and participants will wear digital devices that monitor physical activity, sleep patterns and blood pressure. The aim is to predict and prevent disease. If successful, the study will expand to include 100,000 subjects [70].

The study suggests that the predictive and personalized medicine based on MLDMs will become a reality. From an idealized perspective, a global description of MLDMs for all diseases and relevant cell types would lead to increased understanding of the relationships between pathogenic mechanisms and disease phenotypes. This would include understanding of comorbidity and subgroups. An important clinical use would be diagnostic reclassification of diseases, which in turn could contribute to more effective diagnosis, drug development and treatment. The next natural aim would be to include a time axis in the reclassified diagnostic disease map. In such a map, diseases should be staged by defining MLDMs at different time points. Ideally, such staging should extend to early and even presymptomatic stages. If so, this could help to identify markers that aid in the prediction and perhaps prevention of disease before it becomes symptomatic. The identification of early and presymptomatic MLDMs based on clinical data would be a very large undertaking that would require population-based studies where the subjects are followed for several years. Alternatively, it could be possible to infer early MLDMs based on analyses of animal models of diseases or in human cells exposed to known external disease triggers, such as T cells exposed to allergen. The clinical advantages of predictive and preventative medicine can be exemplified by early treatment of rheumatoid arthritis and multiple sclerosis, which reduces the risk of debilitating disease [71]. If these examples can be generalized, medicine would be likely to change from reactive to proactive.

Clinical research is rapidly entering the era of low-cost personalized omics, and we believe that systems medicine is ideally placed to make sense of this sea of complex data, resulting in tangible improvements in patient care and treatment.

## Abbreviations

GWAS: Genome-wide association study
MLDM: Multilayer disease module
PPI: Protein-protein interaction
SNP: Single-nucleotide polymorphism
siRNA: Short interfering RNA
TF: Transcription factor
